# Trophic distribution of nutrient production in coral reef fisheries

**DOI:** 10.1098/rspb.2023.1601

**Published:** 2023-10-04

**Authors:** James P. W. Robinson, Emily S. Darling, Eva Maire, Mark Hamilton, Christina C. Hicks, Stacy D. Jupiter, M. Aaron MacNeil, Sangeeta Mangubhai, Tim McClanahan, Yashika Nand, Nicholas A. J. Graham

**Affiliations:** ^1^ Lancaster Environment Centre, Lancaster University, Lancaster LA1 4YQ, UK; ^2^ Wildlife Conservation Society, Global Marine Program, Bronx, NY 10460, USA; ^3^ Department of Ecology and Evolutionary Biology, University of Toronto, Toronto, Ontario, Canada; ^4^ Melanesia Program, Wildlife Conservation Society, 11 Ma'afu St, Suva, Fiji; ^5^ Ocean Frontier Institute, Department of Biology, Dalhousie University, Canada B3H 4R2; ^6^ Australian Institute of Marine Science, Townsville, Queensland, Australia

**Keywords:** trophic, seafood, productivity, fisheries, biomass, coral reef

## Abstract

Coral reef fisheries supply nutritious catch to tropical coastal communities, where the quality of reef seafood is determined by both the rate of biomass production and nutritional value of reef fishes. Yet our understanding of reef fisheries typically uses targets of total reef fish biomass rather than individual growth (i.e. biomass production) and nutrient content (i.e. nutritional value of reef fish), limiting the ability of management to sustain the productivity of nutritious catches. Here, we use modelled growth coefficients and nutrient concentrations to develop a new metric of nutrient productivity of coral reef fishes. We then evaluate this metric with underwater visual surveys of reef fish assemblages from four tropical countries to examine nutrient productivity of reef fish food webs. Species' growth coefficients were associated with nutrients that vary with body size (calcium, iron, selenium and zinc), but not total nutrient density. When integrated with fish abundance data, we find that herbivorous species typically dominate standing biomass, biomass turnover and nutrient production on coral reefs. Such bottom-heavy trophic distributions of nutrients were consistent across gradients of fishing pressure and benthic composition. We conclude that management restrictions that promote sustainability of herbivores and other low trophic-level species can sustain biomass and nutrient production from reef fisheries that is critical to the food security of over 500 million people in the tropics.

## 1. Introduction

Measuring the structure and composition of ecological communities provides insights into how energy and nutrients flow through food webs [1,2], and how these processes support ecosystem services to society. Many aquatic ecosystems provide services through fisheries [3], such as nutrition, food security and coastal livelihoods, that can vary regionally in response to interacting human and environmental drivers, and social–cultural contexts. Our understanding of variation in ecosystem services has developed, in part, through large-scale comparative studies of community structure along human and environmental gradients [4,5], helping to uncover fishing-induced biomass depletion [6,7] and biodiversity declines [8].

Such ‘space-for-time’ analyses are particularly informative in the tropics, where highly diverse ecological communities provide essential ecosystem services, but management is often data-limited. For example, coral reefs support important local food systems for an estimated 500 million people worldwide [9], and much of our understanding of how coral reef fishes contribute to fisheries is based on ecological surveys that measure fish biomass at one point in time in multiple places. Large-scale comparative analyses of these datasets have revealed how fish assemblage composition changes along gradients in fishing pressure [10–12] and abiotic processes (e.g. temperature [13]). However, fish biomass is a static snapshot of a coral reef assemblage that fails to capture the growth of targeted populations [14,15] responsible for the rate of biomass production and turnover over days and years [16]. Analyses of fish biomass alone can also overlook socially important aspects of reef fisheries, such as the nutrient concentration of fisheries catches [17]. Considering large-scale associations between production of biomass and nutrients, and how these processes vary between fish species, will more accurately capture potential fisheries service contributions to tropical food systems and inform management of fisheries under pressure from climate change and other anthropogenic impacts.

In practice, both growth rate and nutrient concentration determine the quantity and quality of food production from coral reefs, but associations between species-level productivity and nutrient concentration remain unclear. Recently, empirical models have been developed to predict growth rates [18] and nutrient concentrations of diverse reef fish species [3,17], providing insights into production of nutritious food on coral reefs. For example, population turnover in smaller, targeted species can increase at moderate fishing levels, buffering biomass depletion [19], while growth in herbivore populations rich in iron and zinc can maintain the nutritional value of reef catch following climate disturbances [17]. Reef fish productivity captures the rate at which biomass is produced by an individual fish (i.e. somatic growth), is predictable for any reef fish species, and when combined with abundance and size survey data allows estimates of assemblage-level biomass production [14,18]. Similarly, nutrient models use ecological and environmental trait information to predict the concentration of essential dietary nutrients contained in fish muscle, and can be combined with species' biomass (or catch) data to estimate the nutrient availability (or yield) for fisheries, providing information on the nutritional quality of reef seafood [17].

Growth rates have been combined with elemental stoichiometry to model carbon and nitrogen flux in reef fish [20], suggesting that productivity and nutrient models could be similarly combined to estimate nutrient production rates in reef fishes. Strong effects of size, diet and feeding categories on growth rate [18] and nutrient concentration [3] suggests that nutrient productivity on coral reefs is likely governed by trophic structure. As such, analysis of nutrient productivity among reefs that vary in benthic composition and fishing pressure should help improve understanding of how changes in the reef food web might impact the availability of nutritious catch for fisheries.

Here, we combine size-based growth models with trait-based nutrient models to estimate the nutrient productivity of coral reef fishes from standard biomass surveys. We use established predictive frameworks to estimate growth coefficients (*K*_max_, rate at which each species approaches its theoretical maximum size [18]) and concentrations of six nutrients essential in human diets (calcium, iron, selenium, zinc, vitamin A and omega-3 fatty acids) for 541 fish species observed on coral reef surveys in Belize, Fiji, Madagascar and Solomon Islands. Our new metric of nutrient productivity combines ecological and fisheries theory with aspects of food systems and human health to understand supply of nutritious seafood from coral reef-associated fisheries. Using underwater visual census data from 333 reef surveys, we assess fishing and benthic drivers of three fishery services: standing biomass, biomass turnover and nutrient production. Surveys were conducted on reefs spanning 19 kg ha^−1^ to over 5000 kg ha^−1^ of fishable biomass, including no-take areas and areas under fisheries restrictions, that varied substantially in benthic composition (hard coral, turf algae, macroalgae and rubble). We used Bayesian multivariate models to quantify fishing and benthic drivers of the trophic distribution of three key fishery services, and use these models to provide management recommendations and insights into fisheries supported by future reef habitats.

## 2. Methods

### (a) Underwater surveys

Coral reefs were surveyed at 320 sites between 2016 and 2020 in four countries spanning three marine ecoregions (Tropical Atlantic: Belize; Western Indian Ocean: Madagascar; Southwest Pacific: Fiji, Solomon Islands). Reefs included areas without fisheries regulations (open-access in Madagascar) and, in all four countries, those with partial fisheries management (e.g. time and area closures, gear and access restrictions) and no-take zones. 22 sites were surveyed in Belize (2019, 2020), 168 sites in Fiji (2016–2019), 75 sites in Madagascar (2015, 2016, 2020) and 59 sites in the Solomon Islands (2016, 2018, 2019). Some sites were surveyed in multiple years, such that the total number of reef surveys was 333. Fish were surveyed using belt transects (5 × 50 m in 79% of surveys, 10 × 50 m in 14% of surveys, 2 × 30 m in 7% of surveys), for 1–8 transects at each site (median replicates = 3). Countries with the highest replication (Belize, 5–8 transects) had smaller transect areas (92% of sites = 60 m^2^), whereas countries with the lowest replication (Madagascar, 1–3 transects) had the largest transects (greater than or equal to 250 m^2^). On each transect, fish were sized to the nearest cm (Belize, Madagascar) or in 5 cm bins (up to 40 cm, then nearest cm; Fiji, Solomon Islands), identified to species-level, and enumerated. We converted fish lengths to mass using published length–weight relationships [21], and estimated the biomass (kg ha^−1^) of each observed fish. We excluded fish less than 5 cm in length, damselfish species that are not targeted in fisheries, and highly mobile elasmobranch species that are difficult to survey accurately [22]. Benthic surveys were conducted during fish surveys using point intercept transects, with benthic taxa identified at every 50 cm point along a 50 m transect line. All surveys were conducted by the Wildlife Conservation Society and archived on the MERMAID online data platform (https://datamermaid.org/).

### (b) Nutrient concentrations of fish tissue

Nutrient content in fishes was predicted by phylogeny and multiple ecological traits, including body size, feeding pathway, trophic level and habitat use [3,17]. These nutrient predictions have been statistically robust, and have enabled nutrient concentrations and yields to be estimated from survey and fisheries landings data to address a diversity of questions related to fisheries and human health [23,24]. We predicted the concentration (100 g^−1^) of calcium, iron, selenium, zinc, vitamin A and omega-3 fatty acids in the raw muscle tissue of each reef fish species, using a hierarchical Bayesian model, available on Fishbase [3,21]. We then used information on recommended nutrient intakes [25] to estimate the nutrient density of each species [26,27], defined as the contribution of one 100 g fillet portion to recommended daily intakes, summed across all six nutrients, for adult women (18–65 years old). The contribution of each nutrient is capped at 100%, thus preventing highly concentrated nutrients (e.g. selenium) from dominating nutrient density values.

### (c) Fish biomass production

Following [14], we estimated the daily productivity of each individual reef fish, using standardized species' growth coefficients (*K*_max_) derived from a meta-analysis of reef fish growth curves [14,18]. This empirical framework has been used to quantify fish productivity in data-limited coral reefs around the tropics, advancing understanding of fishing effects [19], energy flux [28] and ecosystem functioning [29]. *K*_max_ is the growth coefficient of the von Bertalanffy growth equation, representing the potential growth trajectory of an individual fish towards its species' maximum size, that can range between 0.011 and 16.43 [18]. Using data and model structure in [18], we predicted *K*_max_ using species' maximum lengths (*L*_max_) and trophic groups for each of the 541 species observed in underwater surveys. In total, 371 species (66% of total species) were out-of-sample predictions using published sources for *L*_max_ [21] and diet group [30]. We then estimated the daily somatic growth (cm) of each individual fish surveyed, according to its observed size (body length) and species-level *K*_max_. Daily somatic growth in length was converted to daily growth in mass using published length-weight coefficients [21]. Therefore, this procedure estimated the daily biomass production potential of each fish observation, which we used as the basis for estimating potential nutrient productivity. Consequently, our analyses focused on a snapshot of the maximum (potential) daily productivity of reef fishes, excluding effects of natural mortality and fisheries exploitation.

### (d) Fishery services

We combined daily productivity estimates with nutrient concentrations to estimate the daily nutrient production of the reef fish assemblage at each site. Specifically, nutrient productivity was the daily productivity of each observed fish multiplied by its edible portion (average value for finfish 87%, [31]) and nutrient concentration, thus representing the maximum daily potential production of nutrients contained in edible, muscle tissue of reef fish, estimated for each of the six nutrients. At each transect, for each trophic group, we estimated the standing biomass (kg ha^−1^), biomass production (g d^−1^ ha^−1^), biomass turnover (biomass production divided by standing biomass, %) and nutrient production (calcium, iron, zinc: mg d^−1^ ha^−1^, selenium, vitamin A: μg d^−1^ ha^−1^, omega-3 fatty acids: g d^−1^ ha^−1^). Trophic groups were defined according to published schemes and expert knowledge, representing herbivores (scraping detritivores), herbivores (browsing macroalgal feeders), planktivores, omnivores (mixed-diets), sessile invertivores, mobile invertivores and piscivores. We excluded sessile invertivores from all analyses as these species contributed an average 2% of nutrient production and were not targeted in fisheries. Transect-level estimates were averaged to give site-level estimates of standing biomass, biomass turnover and nutrient production of each trophic group, thus reducing sampling variability arising from the number and size of transects. These metrics describe three fisheries services, representing catch available to fishers (e.g. fishable biomass), long-term catch turnover (e.g. biomass production and turnover) and the potential contribution of reef fish to diets through fisheries (e.g. nutrient production). We then converted these estimates into relative contributions of each trophic group to each fishery service (%), which we use as a representation of fish assemblage trophic structure.

### (e) Drivers of fishery services

We developed statistical models to assess the drivers of fishery service trophic structure: the relative contributions of three functional fish groups targeted by fishers (herbivores, mobile invertivores, piscivores) to biomass, biomass turnover and nutrient productivity. As such, we recalculated contributions for each group, combining browsing and detritivorous herbivores into one group (herbivores), for each reef site (*n* = 333). We also estimated the mean per cent cover of five major benthic groups at each site (hard coral, turf algae, macroalgae, rubble, bare substrate). These estimates were fitted to Bayesian models with Dirichlet distributions, using fixed covariates of total fishable biomass (kg ha^−1^), benthic cover (hard coral, turf algae, macroalgae, rubble, bare substrate), and depth (m). To capture potential for different fishing (e.g. selectivity, gear, effort) and environmental effects (e.g. upwelling, primary productivity) in each country, we fitted country-level biomass effects (i.e. varying slopes). Management regime was included as a group-level intercept nested with country.

All continuous variables were centred with a mean of 0 and scaled by dividing each variable by its standard deviation. Models were implemented in brms [32] and sampled in Stan, using R v4.2.0 [33]. We sampled four chains with 3000 iterations each, and ensured model convergence by inspecting divergent transitions and ensuring that Rhat was less than 1.01. For each fishery service, model posteriors were sampled to estimate the median posterior trophic structure at each reef (proportion of herbivore, mobile invertivore and piscivore). We used these estimates to quantify reef trophic pyramid structure, where reefs with greater than 50% contributions from herbivores were bottom-heavy and reefs with less than 50% contributions from herbivores were top-heavy. For nutrient productivity, we also generated out-of-sample predictions of trophic contributions from herbivores, mobile invertivores and piscivores along fishable biomass gradients in each country.

## 3. Results

### (a) Associations between growth rate and nutrient concentration

**Figure 1.**
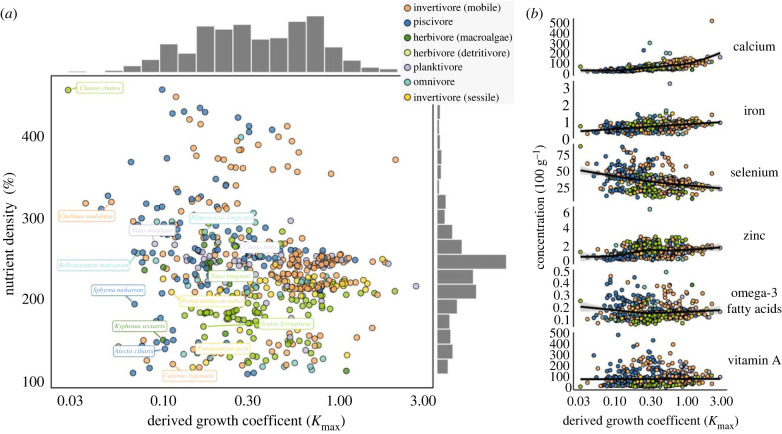
Association between nutrient content and growth potential of 541 coral reef fish species. Points are individual species observed on underwater visual census surveys conducted in Belize, Fiji, Madagascar and the Solomon Islands, and *K*_max_ is plotted on a log scale. (*a*) Nutrient density is the combined contribution to recommended daily women intakes of calcium, iron, selenium, zinc, omega-3 fatty acids and vitamin A [27], using reference values for adult women (18–65 years old). (*b*) The six nutrient concentrations by *K*_max_ for each species, with fitted GAM smoothers (± 95% confidence interval). Growth coefficient *K*_max_ is the value of growth coefficient *K* for each species at its theoretical maximum size, derived from the von Bertalanffy equation [18]. Labels show the top two species with highest average biomass in the dataset, for each trophic group, and marginal histograms show data distributions on each axis.

Most reef fishes had nutrient concentrations that met recommended intakes of two to three nutrients in a 100 g portion (nutrient densities between 90 and 250%), including species with ‘slow' or ‘fast' growth coefficients (*K*_max_ between 0.06 and 2.8). Species with the highest nutrient densities (greater than 300%) were mostly piscivores and mobile invertivores, including slow-growing species such as snappers (Lutjanidae) and groupers (Epinephelidae) with lower *K*_max_ values of 0.3 (figure 1*a*). The fastest growing species (*K*_max_ > 1) were dominated by mobile invertivores (14 species), sessile invertivores (10) and planktivores (6), most of which had nutrient densities between 200 and 250%, while herbivores were generally less nutritious, with intermediate growth rates (figure 1*a*). Nutrient density and *K*_max_ were weakly associated (*r* = −0.1), but nutrient density obscured associations between *K*_max_ and concentrations of specific nutrients. For example, nutrients that vary strongly with body size [3] (electronic supplementary material, figure S1) were more strongly correlated with *K*_max_, which also varied with size. As a result, growth–nutrient relationships were positive for calcium, iron, and zinc, and negative for selenium (figure 1*b*). By contrast, omega-3 fatty acid and vitamin A concentrations were not associated with *K*_max_.

### (b) Fishery services: standing biomass, biomass turnover and nutrient production

**Figure 2.**
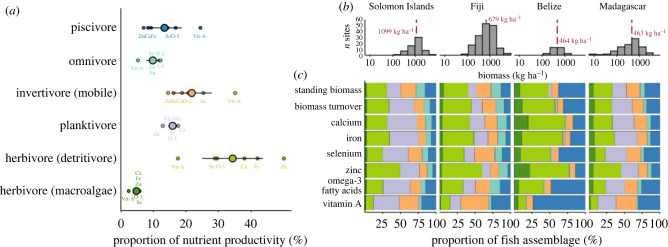
Nutrient productivity of fish trophic groups. (*a*) Mean contribution of each trophic group to site-level nutrient production for each nutrient (small labelled points) and the average across nutrients (large point ± 2 s.e.m.) (by country in electronic supplementary material, figure S3). (*b*) Histograms show log_10_ biomass distribution across sites in each country (red dashed line is median biomass). (*c*) Bars show the average contribution of each trophic group to standing biomass, biomass turnover and nutrient productivity of six nutrients, by country.

Species-level differences in growth rates and nutrient concentrations (figure 1) may not necessarily scale up to influence assemblage-level nutrient production, which is also governed by species' relative abundances. Next, we estimated standing biomass (kg ha^−1^), biomass turnover (productivity, kg ha^−1^ d^−1^, divided by standing biomass, %), and nutrient production (mass of nutrients assimilated in fish tissue d^−1^ ha^−1^) by the reef fish assemblage at 333 sites in Belize, Fiji, Madagascar and the Solomon Islands. Reefs supported a range of biomass levels (9 kg ha^−1^ in one Madagascar site to 5937 kg ha^−1^ in one Fiji site, figure 2*b*), and biomass production generally increased with fishable biomass (electronic supplementary material, figure S2). Biomass turnover, however, was highly variable along the biomass gradient (1–41%), while reefs in Fiji and the Solomon Islands had the highest nutrient productivity (at approx. 2000 kg ha^−1^ of fish biomass), particularly minerals (calcium, iron, selenium, zinc) (electronic supplementary material, figure S2). These fishery services were provided by different fish trophic groups, with the herbivore (detritivore) fishes dominating nutrient production (mean = 34% across all six nutrients, ranging from 18% to 50%). Mobile invertivores were the second highest nutrient producer, with an average of 22% of the production across all nutrients and accounted for more vitamin A production (35%) than herbivores (detritivore) (18%) (figure 2*a*). Other trophic groups had lower contributions to nutrient production, contributing a mean 16% (planktivore), 13% (piscivore) and 10% (omnivore).

Herbivores (detritivore) and mobile invertivores were therefore the largest contributors to fishery services, accounting for an average 56% of standing biomass and biomass turnover, and 40–69% of nutrient production (figure 2*c*). However, trophic group contributions varied between countries (electronic supplementary material, figure S3). For example, piscivores were the largest contributors to standing biomass and omega-3 and vitamin A production in Belize, whereas planktivores dominated biomass turnover and production of most nutrients in Solomon Islands (figure 2*c*). Zinc production was dominated by herbivores (detritivore) in all countries, likely because this nutrient is more concentrated in low-trophic species.

### (c) Bottom versus top-heavy fishery services

**Figure 3.**
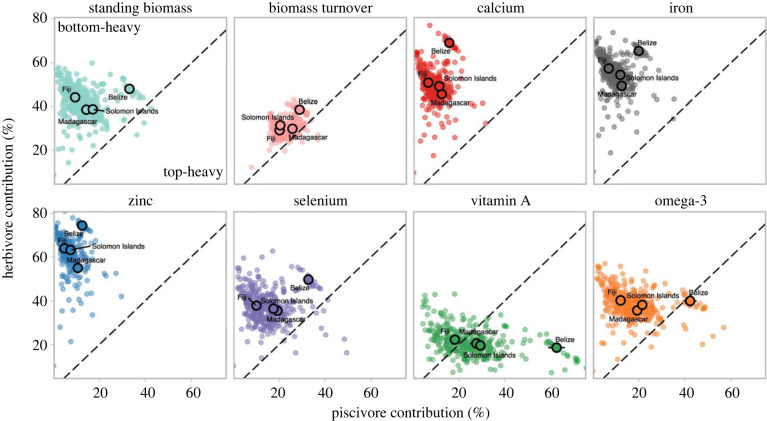
Contribution of herbivores and piscivores to fishery services. Points show the posterior median predicted herbivore and piscivore contribution (%) to each fishery service, for all 333 reef surveys. Bolded points represent the mean values in each country (± 2 s.e.m.). Points in the upper-left have bottom-heavy pyramids (greater contributions from herbivores than piscivores) and points in the bottom-right have top-heavy pyramids (greater contributions from piscivores than herbivores). S.e.m. is provided in electronic supplementary material, table S1.

We next fitted multivariate composition models to understand drivers of trophic group contributions to fishery services. We focus on herbivores (detritivores and macroalgal-feeders combined), mobile invertivores and piscivores because all are typically targeted by fisheries, and simplified this multivariate trophic structure by defining reefs as bottom-heavy when the relative biomass of herbivores exceeds piscivores, and top-heavy when piscivores dominate over herbivores. Standing biomass, biomass turnover and productivity of five nutrients (calcium, iron, selenium, zinc and omega-3) were bottom-heavy at over 93% of reefs, indicating that herbivores contributed a significant proportion of these fishery services (figure 3). Only two fishing-restricted reefs had top-heavy biomass distributions (in Madagascar), whereas vitamin A production was top-heavy at 60% of reefs (figure 3). Belize had the most top-heavy trophic structure, where piscivores accounted for 42% of omega-3 fatty acid production and 62% of vitamin A production.

Variation in the contribution of fishes to nutrient productivity was only partly associated with differences in fishable biomass. For example, trophic distributions varied between Belize, Fiji and Solomon Islands, but did not change substantially along the biomass gradient within each country (electronic supplementary material, figure S4), suggesting that unmeasured historical processes (e.g. disturbance and fishery dynamics) or biogeographic differences also govern assemblage composition of these reefs. By contrast, nutrient production in Madagascar shifted from dominance by mobile invertivores at low-biomass to herbivores at high-biomass (electronic supplementary material, figure S4). Madagascar's reefs had the lowest biomass in the dataset, suggesting that biomass depletion due to fishing has changed trophic structure. In all four countries, nutrient production from piscivores increased with fishable biomass but, at most mid- and low-biomass reefs, piscivores accounted for less than 10% of nutrient production. However, the management regime had weak and uncertain effects on the relative contribution of fish groups (electronic supplementary material, figure S5), with no-take areas and partially managed areas (e.g. access or gear restrictions) having similar trophic group contributions within each country (accounting for benthic effects). Only Madagascar had reefs that were openly fished, but these were similar in fish composition to no-take and restricted reefs.

Benthic composition also influenced which fish groups contributed to nutrient production. Coral cover ranged from 0 to 84%, with low-coral-cover reefs characterized by dominance of macroalgae (Belize, Fiji, Madagascar), rubble (Fiji, Solomon Islands) or turf algae (Solomon Islands) (electronic supplementary material, figure S6). Nutrient contributions from herbivores increased with hard coral and macroalgae cover, while mobile invertivores produced relatively more calcium and vitamin A as rubble increased (electronic supplementary material, figure S7). Piscivores produced relatively fewer nutrients on reefs with more bare substrate, and more vitamin A and omega-3 fatty acids on deeper reefs (electronic supplementary material, figure S7). These opposing benthic trends influenced the pyramid shape of fishery services. For example, only the deeper reef survey locations (greater than 14 m, Belize and Madagascar) had top-heavy pyramids for standing biomass and biomass turnover, while reefs dominated by either coral or macroalgae (greater than 60% cover) only supported bottom-heavy pyramids for all fishery services (electronic supplementary material, figure S8).

## 4. Discussion

Empirical analysis of trophic distributions can help to delineate the structure and function of coral reef food webs, revealing ecological rules [2], environmental forcing [13] and human impacts [10]. Yet coral reef fish assemblages have largely been described using static measures of ecosystem state (e.g. fish biomass), potentially obscuring contributions from lower trophic levels to ecosystem productivity [16,28] and fisheries catch [34]. Here we combined species-level growth rate and nutrient concentrations with underwater surveys to show that herbivores and mobile invertivores dominate trophic structure on coral reefs, which is consistent with theoretical expectations [2] and previous empirical studies [10,35]. We also showed that biomass turnover and nutrient production by fishes are more bottom-heavy in trophic pyramids than fishable biomass, further underlining the importance of lower trophic levels in channelling benthic production and nutrients through reef food webs that in turn can support productive and nutritious coastal fisheries under sustainable management.

At the species-level, nutrient density and biomass production were weakly associated, but this was partly because nutrient density (an aggregate metric of nutrient concentrations) obscured relationships between growth rate and concentrations of individual nutrients. Empirical models show that reef fish growth rates are fastest in small-bodied species [36,37] and, on average, higher in herbivores and piscivores [18], whereas nutrient concentration varies predictably with body size and traits such as trophic level and diet [3,17]. We found that size-linked nutrients such as calcium, iron and zinc were more concentrated in low-trophic-level species with fast biomass turnover, possibly reflecting dependence on energy pathways that are more concentrated in these minerals (e.g. benthic or detrital energy versus pelagic) [38]. Higher-trophic level species, by contrast, integrate energy across multiple energy pathways (pelagic, benthic, detrital) [39,40], likely dampening nutrient concentrations. For example, slow-growing species living at depth had greater selenium concentrations, possibly reflecting foraging in deeper reef habitats associated with higher selenium content in marine fishes globally [3]. Omega-3 fatty acids and vitamin A concentrations, however, had weak associations with species' potential biomass turnover, indicating that growth rate is a poor predictor of these nutrients in reef fishes.

Species-level nutrient and growth rate values must be combined with abundance or biomass data to understand nutrient flux and productivity at the scale of reef fish assemblages [20]. We integrated growth rate, nutrients, and biomass to assess the contribution of fish trophic groups to three fishery services provided by coral reefs (standing biomass, biomass turnover and nutrient production). Fishery services were dominated by herbivores (i.e. bottom-heavy) at most reefs, but the relative contribution of trophic groups to fishery services also varied regionally, between Pacific (Fiji, Solomon Islands), Indian Ocean (Madagascar) and Caribbean (Belize) reefs. In Belize, for example, browsing herbivores had the highest biomass, likely because macroalgae was present at all reefs (electronic supplementary material, figure S6), suggesting that fishery services at macroalgae-dominated reefs are dominated by browser species. Fiji had the most bottom-heavy trophic pyramids, suggesting that these reefs have particularly high benthic productivity, with both algal and coral regimes supporting high biomass turnover of herbivorous (scraping detritivore) species. These regional differences are likely linked to abiotic processes that constrain energy and nutrient flux through reef food webs (e.g. temperature, irradiance, upwelling) [13,16], disturbance history (e.g. fishing, thermal stress) [41] and intrinsic regional differences in benthic and fish community composition. Analyses that quantify abiotic influences on benthic and pelagic primary production, fishing intensity on different trophic groups, and energy flux through food webs are required to fully understand regional variability in coral reef trophic structure. Such assemblage-level analyses of rate-based ecosystem metrics (e.g. productivity) will help to inform understanding of general patterns in the structure and composition of food webs [42].

Analyses of coral reef trophic structure have largely focused on biomass gradients [10,11,35], but here we also assessed links between benthic composition and the relative abundance of fish trophic groups. Surveyed reefs supported a mix of coral, rubble and algae-dominated habitats (electronic supplementary material, figure S6), and bottom-heavy trophic pyramids were also prevalent in all of these benthic regimes. These sites have experienced recent disturbances to reef habitat, such as cyclones and coral bleaching, but these did not appear to substantially change trophic group contributions to fishery services. Such modelling of habitat drivers of fish trophic groups can provide information for adapting management to future reefscapes. For example, we found that shifts to rubble dominance increased the contribution of mobile invertivores to nutrient production, consistent with increases in goatfish (Mullidae) populations after coral declines [43]. Gears targeting these species could help maintain nutritious catches for people from fisheries after coral mortality events, but this should be informed by knowledge on which species groups are preferentially targeted by fishers, and thus likely to be consumed locally. By contrast, nutrient production from reef herbivores increased with coral and algal cover, adding further evidence that herbivores are likely to play a key role in supporting food security on both coral-dominated and degraded reefs [17,28,44].

### (a) Managing trophic structure of fishery services

Contributions to nutrient productivity remained relatively constant along biomass and trophic group gradients in Belize, Fiji and Solomon Islands, and between protected and partially managed reefs (electronic supplementary material, figure S5). These patterns are similar to those observed on Indian Ocean [35] and Indonesian reefs [11], where most reefs were dominated by invertivores and herbivores, but fish composition shifts from convex (dominated by mid-trophic levels) to concave (dominated by low and high trophic levels) trophic structures as community fish biomass increases [10]. However, fishing levels that deplete fish biomass below 100 kg ha^−1^ can release benthic invertebrate populations such as sea urchins, promoting dominance of invertebrate energy pathways in reef food webs [10]. Surveyed sites in Madagascar had the lowest total biomass (less than 100 kg ha^−1^ of fishable biomass), suggesting these reefs likely experience very high levels of fishing pressure. On these low-biomass reefs, mobile invertivores replaced herbivores as the dominant nutrient producers, suggesting these food webs are primarily supported by invertebrate energy pathways [45], creating losses in potential fisheries catch from herbivorous species.

Reefs in Belize, Fiji and Solomon Islands had fishable biomass above 100 kg ha^−1^ and also maintained trophic structure across their biomass gradient. Such consistency mirrors findings from a recent global reef analysis [16], indicating that regulating fishing to avoid biomass depletion can be an effective method of protecting fish trophic structure. We note that 100 kg ha^−1^ is likely to be an extreme biomass depletion, below the 300–600 kg ha^−1^ that is recommended to avoid fishery collapse [35]. Yet in the four countries analysed here, most reefs were managed using diverse fishing regulations (e.g. gear and access restrictions, area and time closures), and most reefs thus likely experienced moderate to high fishing effort, suggesting that all management forms can be effective in protecting the trophic composition of fishery services. Indeed, no-take areas had similar trophic structure to ‘partially' managed reefs—evidence that both conservation and fishing goals can be achieved through gear and area restrictions [11], whereas open-access reefs (Madagascar) experienced extreme biomass depletion and disrupted trophic structure. However, analyses of trophic composition may mask shifts in the species that provide most fishery services. Species composition typically responds strongly to fishing, with high fishing pressure associated with shifts in catch composition [46] and diminished functioning, if key species become depleted (e.g. excavating parrotfish) [47].

Such alignment of conservation and fishing goals is particularly relevant for herbivorous scraping and browsing species that are targeted in fisheries across the tropics [48] but also have key functional roles in promoting coral settlement through grazing of detritus and algae on reef substrate [49]. Maintaining sustainable fishing of herbivore populations while protecting ecosystem functioning is thus a central challenge for fisheries management on coral reefs [50,51]. Our results suggest that ‘partial' fishing management in Belize, Fiji, Solomon Islands successfully protects contributions of herbivorous fishes to biomass production. Though we were unable to assess herbivory in this study, long-term research in Kenya suggests that herbivore populations can experience light exploitation and continue to exert grazing pressure on reef substrate [52], provided fishing effort is regulated above biomass thresholds [35] and large-bodied herbivorous fishes have sufficient time to recover from biomass depletion [53,54]. However, grazing pressure on coral reefs is context-dependent (e.g. effects of depth, biodiversity, fish behaviour) [55–57], and can be decoupled from herbivore biomass production [29], which may explain the presence of fleshy algae in all four surveyed countries. Research incorporating metrics of grazing functions and fishery catch will help managers to balance potential trade-offs between ecosystem services provided by herbivores.

Despite implementation of fishery restrictions at most reefs, piscivores were rarely observed. Correspondingly, piscivores generally had minor contributions to biomass turnover and nutrient production, despite these species having high nutrient density. Top-heavy fishery services (i.e. dominated by piscivores) were only observed for vitamin A in Belize or on reefs with high fishable biomass. These patterns underline the diminished functional importance of piscivores in fished seascapes and small protected areas [10,35,58,59], suggesting that these species contribute less than other reef fishes to fisheries catch or tropical seafood supply, even on lightly fished reefs. Such findings highlight the importance of trade-offs and associations between biomass, biomass production and nutrients.

Further research on the contributions of trophic groups to fishery services should integrate ecological surveys from other habitats. Specifically, using catch composition data to help ensure key non-reef stocks are surveyed alongside coral reef fishes. Small-scale coastal fishers also target fishes in habitats connected to coral reefs (e.g. seagrass, mangrove) [60,61] that were not included in this analysis, and both fisher effort and species selectivity can vary spatially on reefs [62]. For example, small-scale fishers in Western Province, Solomon Islands target up to 382 species, but only 56% of these were observed in these reef surveys, leaving 216 species either not observed on reefs or likely caught in other habitats (e.g. pelagic fish: Carangidae, Corhyphaenidae). Nutrition-sensitive fisheries management, which prioritizes catch of nutrients relevant for local diets, rather than biomass, could therefore focus on regulating herbivore and mobile-invertivore fisheries that produce the majority of biomass and nutrients. Indeed, gear-based management is already effective at reducing capture of rare, non-target species [63]. Such approaches can now be combined with predicted nutrient concentrations to recommend gears that supply long-term sustainable catch, maximizing nutrient yields for people consuming reef seafood [23].

We used statistical models fitted to published data to make predictions of nutrient concentrations in reef fish species, and combined these with ‘snapshot' fish surveys that capture community size structure and species composition, both of which are spatially and temporally variable. These steps were necessary to estimate assemblage-level nutrient productivity among diverse and data-limited reef fishes, but we note that scaling underwater snapshots of reef fish communities to dynamic processes (e.g. nutrient productivity) remains a fundamental challenge for coral reef science [64]. Confronting empirical analyses of underwater surveys with alternate approaches, such as ecosystem models, will help to assess strengths and limitations of scaling individual fish to dynamic ecosystem properties. Nevertheless, growth rate [18] and nutrient content models [17] have been validated for statistical performance on reef fishes, and their predictions have been effectively combined with ecological surveys to update our understanding of the structure and functioning of coral reefs [29,65].

### (b) Future directions

Our study provides a framework for estimating nutrient productivity from fish survey data using publicly available statistical predictions of fish growth rates [14] and nutrient concentrations [21]. Nutrient productivity measures the turnover of nutrients between trophic levels, offering insights into nutrient flux in reef food webs and providing a new approach for quantifying food provisioning from coral reefs. This rate-based metric unites concepts from food systems, ecology and fisheries theory, and is complementary to emerging research on reef fish productivity [16,19,28] and elemental flux [20]. In addition to refining growth rate and nutrient estimates and their application to snapshot UVC, future research will need to consider potential for environmental variation to influence nutrient concentrations in fish [17,66], and how such intraspecific variation might have knock-on effects for nutrient flux through reef food webs.

We analysed trophic groups of fish with similar diets and behaviours that are relevant to fisheries (herbivores, piscivore, mobile invertivores) [11], whereas previous large-scale analyses delineated reef fish pyramids using trophic levels [10,16]. Since fisheries management typically focuses on species or gear restrictions that affect catch selection, we suggest regulating fishing using trophic groups (versus trophic levels). This approach also avoids issues arising from assigning a single trophic level to species with diverse (e.g. ‘nominal' herbivores [67]) and variable diets (e.g. ontogenetic shifts [68]). Our analysis also focused on reef fish species observed in ecological surveys, but not invertebrates that contribute to energy and nutrient flux in coral reef food webs [69], particularly in rubble habitats. Analysis of entire food webs (i.e. fish, invertebrates and primary producers) is a longstanding challenge in coral reef science, owing to high turnover of small, cryptic species [36] and high biomass of mobile top predators [13], both of which are difficult to census accurately at comparable spatio-temporal scales. Our results suggest that invertebrate biomass (or population turnover) was higher on rubble and low-biomass reefs that supported the largest (relative) biomass of mobile invertivores. Indeed, invertebrates can be significant contributors to energy flux in reef food webs [69,70], while many reefs support invertebrate fisheries that may benefit vulnerable people (e.g. reef gleaning by women) [71]. Analysis of invertebrate contributions to coral reef biomass, biomass turnover and nutrient production, and better integration of ecological and fisheries surveys with nutritional values for invertebrates, will help to address these knowledge gaps.

## 5. Conclusion

Our analysis of coral reefs in four countries spanning the tropics showed the dominance of low trophic-level fishes in standing biomass, biomass production and nutrient production. Coral reef herbivores are likely to be the primary contributor to ecosystem and fisheries services across diverse (and disturbed) benthic habitats, fisheries management strategies and at reefs with varying biomass levels, underlining their importance for tropical food security. Here, reefs that avoided extreme biomass depletion (i.e. greater than 100 kg ha^−1^) maintained herbivore dominance, affirming the potential for fisheries management to reach biomass thresholds [35] and nutritious fisheries catches. Higher biomass thresholds (300–600 kg ha^−1^) are closer to biomass-based multispecies maximum sustainable yields, and thus will have additional benefits for ecosystem-level sustainability targets, as shown in large-scale analyses of both fished and remote reefs [11,35,72]. We also found that the trophic structure of reef fishery services was resilient to different management strategies (e.g. gear and access restrictions, no-take areas), supporting use of culturally and socially appropriate management that permits fishing. Our framework provides an interdisciplinary approach that integrates theory across ecology, human health and fisheries science, helping focus efforts on protecting and maximizing sustainable seafood supply to food-insecure people [9,73].

## Data Availability

Underwater visual surveys are available from https://datamermaid.org. All data and R scripts required to reproduce analyses are at https://github.com/jpwrobinson/NutrientProductivity. Supplementary material is available online [74].
